# Primary Cutaneous T-Cell Lymphoblastic Lymphoma: Case Report and Literature Review

**DOI:** 10.1155/2019/3540487

**Published:** 2019-02-20

**Authors:** Sharad Khurana, Manuel Beltran, Liuyan Jiang, Ernesto Ayala, Vivek Roy

**Affiliations:** Mayo Clinic, Jacksonville, FL, USA

## Abstract

Cutaneous involvement by precursor T-cell lymphoblastic leukemia/lymphoma (T-ALL/LBL) is rare, and almost all cases are seen in association with bone marrow, blood, and/or lymph node involvement. Presentation with isolated skin involvement is very rare. Literature review revealed only one case report of primary cutaneous T-cell LBL. We discuss here another patient diagnosed with primary cutaneous T-cell LBL at our institute. This patient was initially misdiagnosed as having peripheral T-cell lymphoma NOS. Cytogenetic analysis showed the CDKN2A deletion (−9p21×2) in addition to three intact copies of ABL1 (+9q34). Although she failed multiple lines of intensive chemotherapy, her disease remained confined to the skin. We believe that this presentation of T-LBL is underreported, and many patients are likely misdiagnosed as having high-grade cutaneous T-cell lymphomas. With this case and literature review, we would like to highlight the importance of keeping lymphoblastic lymphoma on the differential diagnosis of cutaneous T-cell lymphoma-like lesions to avoid delay in diagnosis and inappropriate treatment of this aggressive disease.

## 1. Introduction

T-cell ALL/LBL accounts for ∼20% of all cases of ALL and is more commonly seen in adults than children [1]. Cutaneous involvement by T-cell leukemia/lymphoma is a rare occurrence with a reported frequency of around 4.3% [2–4]. We present a patient diagnosed with primary cutaneous T-cell LBL at our institute. This patient was initially misdiagnosed as having peripheral T-cell lymphoma NOS as immunohistochemical staining for immature T-cell markers was not performed at presentation. The patient had an aggressive course with disease relapse and progression after multiple lines of intensive chemotherapy. However, her disease remained confined to the skin. Literature review revealed primary cutaneous T-LBL to be a very rare entity with only one previously reported case [5].

## 2. Case Description

**Figure 1:**
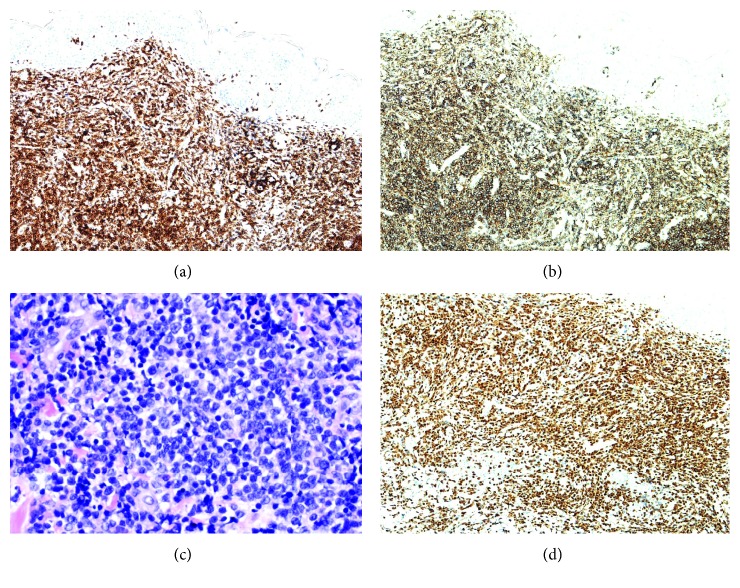
(a) Positive CD3 immunostaining. (b) Positive CD4 immunostaining. (c) H&E stain with blastic appearing lymphocytes. (d) Positive immunostaining with terminal deoxynucleotidyl transferase (TdT) of the lymphocytes.

A 52-year-old woman with past medical history of diabetes mellitus type 2, atrial fibrillation, and hypertension presented with multiple, small, and reddish papular lesions on both lower extremities. She described these lesions as “bug bites” which rapidly progressed in size and number, involving most of her body in a matter of eight weeks. She had no other symptoms at this time. The patient underwent excisional biopsy of one of the lesions which showed diffuse infiltrate by atypical lymphoid cells. Flow cytometry and immunohistochemical studies showed these atypical cells as CD4+ T cells that expressed CD2, CD3, CD5, partial CD57, partial CD52, partial CD26, and alpha/beta receptors (Figure 1). CD7, CD8, and CD30 were not expressed. These atypical lymphocytes had weak and focal expression for BCL2 but negative CD20 and BCL-6 expression. Myeloperoxidase (MPO), CD34, and CD117 were not expressed ruling out myeloid lineage malignancy. The proliferative rate by Ki-67 was moderate at 70%. Polymerase chain reaction (PCR) study for T-cell receptor gamma gene rearrangement was positive and for T-cell receptor, beta gene was oligoclonal. Terminal deoxynucleotidyl transferase (TdT) immunostaining was not performed. No metaphases were available for karyotyping. A final diagnosis of peripheral T-cell lymphoma NOS was made after evaluation by two different pathology centers. The lymphoma was limited to the skin with no involvement of bone marrow and lymph nodes or any extranodal organ/tissue as confirmed by a positron emission tomography (PET-CT). HTLV-1 and HIV blood testing were negative. The patient was treated with six cycles of CHOEP (cyclophosphamide, doxorubicin, vincristine, etoposide, and prednisone) and went into complete clinical remission. Posttreatment PET scan showed resolution of all the metabolically active skin lesions. She was then referred to our institution for consideration of autologous stem-cell transplant as consolidation.

**Figure 2:**
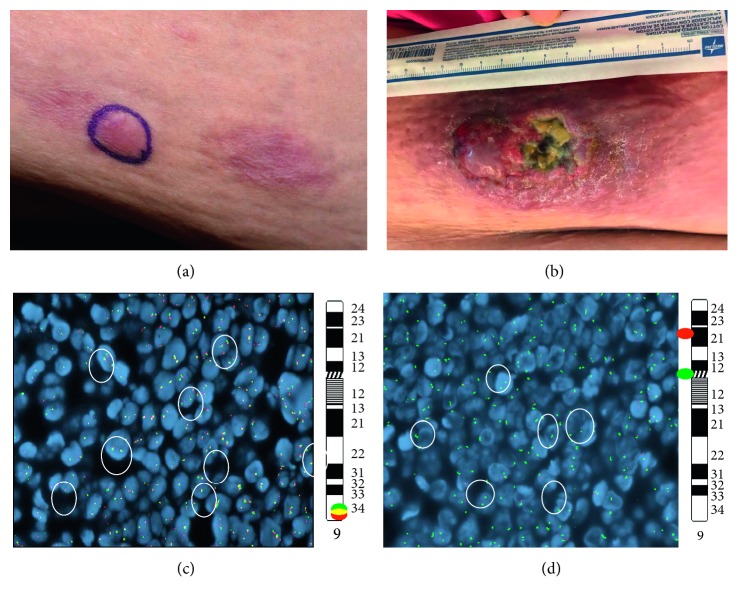
(a) 2 × 1 cm lesion on the right lower quadrant of the abdominal wall at first relapse. (b) Enlarging right lower abdominal wall lesion now almost 15 cm × 11.5 cm with ulceration. (c) Break-apart ABL1 fluorescence in situ hybridization (FISH) circles indicate 3 intact ABL1 signals (5′ABL1 (green)/3′ABL1 (red) {9q34}). (d) Enumeration for 9p-circles indicates homozygous loss of CDKN2A (CDKN2A(9p16) red/D9Z19(CEN) green).

Three months after completion of chemotherapy while undergoing pretransplant evaluation, the patient noticed a slightly raised, erythematous 2 × 1 cm lesion on the right lower quadrant of her abdominal wall (Figure 2). Biopsy of this lesion showed neoplastic lymphocytes, demonstrating a similar immunophenotype as that was seen on the initial skin biopsy. However, both the dermatopathologist and hematopathologist noticed that the morphology of the lymphocytes appear to be blastic in appearance; therefore, immunostain for terminal deoxynucleotidyl transferase (TdT) was performed, which showed diffuse nuclear positivity in the lymphocytes, confirming lymphoblastic nature of these cells (Figure 1). As such, a final diagnosis of T-cell lymphoblastic lymphoma was made. Fluorescence in situ hybridization (FISH) study revealed homozygous CDKN2A deletion (−9p21 × 2) and three intact copies of ABL1 (+9q34) (Figure 2), which aided in confirming the final diagnosis. Subsequent bone marrow biopsy was negative for involvement by T-cell lymphoblastic lymphoma/leukemia. No suspicious foci of increased FDG uptake were noted on the PET-CT of the whole body. We also reviewed the original skin biopsy and performed immunostain of TdT, which was again diffusely positive in the neoplastic lymphocytes. Further, we repeated and compared the PCR studies for TCR gene rearrangement between these two skin biopsies using multiple master mixes target conserved regions within the variable (V) and the joining (J) regions for T-cell receptor gamma gene and conserved regions within the variable (V), diversity (D), and the joining (J) regions for T-cell receptor beta gene. In each biopsy, a clone (amplicon at 264 dp) in the V-J region of beta gene and another clone (amplicons at 201 dp and 220 dp) at the V-J region of gamma gene were identified; and the results appeared to be identical. Therefore, the diagnosis of cutaneous T-cell lymphoblastic lymphoma at initial presentation and at relapse was confirmed.

She was treated with high-dose methotrexate and cytarabine along with prophylactic intrathecal chemotherapy with cytarabine and methotrexate, with the plan of proceeding with allogeneic hematopoietic stem cell transplantation. Unfortunately, her disease progressed after 2 cycles of chemotherapy as evidenced by enlarging right lower abdominal wall lesion, now almost 15 cm wide and 11.5 cm in length with ulceration (Figure 2). PET-CT showed hypermetabolic right lower abdominal wall subcutaneous lesion with associated reactive inguinal and external iliac lymphadenopathy. Repeat skin biopsy confirmed T-LBL, and chromosomal microarray (CMA) analysis performed using molecular inversion probes on a whole genome array showed multiple complex genomic alterations. Therapy was now switched to second-line nelarabine to obtain disease control before proceeding with allogeneic stem cell transplantation. The patient however had no response to nelarabine and her skin lesion kept progressing, for which she was referred to radiation oncology for palliative radiation to the ulcerated enlarging tumor.

## 3. Discussion

Approximately 5,960 new cases and 1,470 deaths attributable to acute lymphoblastic leukemia/acute lymphoblastic lymphoma (ALL/LBL) are estimated in 2018 in the United States of America [6]. T-cell ALL/LBL accounts for ∼20% of all cases of ALL and is more common in adults than children [1]. It accounts for 20% of all cases of leukemia in adults [7] with a 2 : 1 male predominance. Lymphoblastic neoplasms can present as either leukemia or lymphoma forms depending on the extent of the bone marrow (BM) infiltration by the neoplastic cells. Lymphoblastic presentation is more often of T-cell lineage than B cell, with an approximate ratio of 9 : 1 [4].

Lymphoblastic lymphomas comprise around 3.5% to 7% of all skin lymphomas [8]. Cutaneous involvement by T-cell leukemia/lymphoma is a rare occurrence with a frequency of around 4.3% as compared to 16–33% patients with B-LBL/ALL [2–4]. T-LBL usually presents as multiple skin lesions throughout the body as compared to B-LBL which can present as a solitary skin lesion [2, 3, 9]. Almost all cutaneous T-LBL cases reported in the literature are seen in association with bone marrow (T-ALL) and/or mediastinal (most common T-LBL site), lymph node, or extranodal involvement (Table 1). Cutaneous LBLs present as diffuse monomorphous infiltrate located in the entire dermis and subcutis without epidermotropism. Immunophenotyping studies are required to distinguish ALL/LBL from other high-grade cutaneous lymphomas like peripheral T-cell NOS. T-LBL is distinguished from peripheral T-cell lymphoma by the presence of immature T-cell phenotypes such as TdT, CD7, and cytoplasmic CD3.

While great progress has been made in recent years in understanding the biology and genetic makeup/mutations associated with T-cell ALL [10], cytogenetic and molecular abnormalities are not well defined in T-LBL and more so in cutaneous T-LBL due to rarity of the disease. A linkage between abnormal expressions of genes located at 9q34 and 17q22–23 has been described [11]. ZNF79, ABL1, or THRAP1 have been noted in the “lymphoma phenotype” such as in bulky masses in the mediastinum with minimal BM involvement [11]. Schraders et al. described a cohort of 12 cases of T-LBL, showing recurrent genomic aberrations affecting gene loci with known roles in cell cycle regulation. The most frequent genomic abnormality in this cohort, observed in 11 cases (92%), was the deletions of the CDKN2A locus, which encodes two tumor suppressor genes, p16INK4a and p14ARF. Deletions of RB1 (16%), a tumor suppressor gene, duplications of MYB (16%), an oncogene locus, and an amplification of ABL1 were also reported [12]. Vezolli et al. reported trisomy 4 and gain of 1p36.33-p22.1 on microarray-comparative genomic hybridization in early-stage disease and multiple chromosomal alterations in the late-stage of primary cutaneous T-LBL [5]. They suggested gain of 1p36.33-p22.1 to be an interesting marker in PC-T-LBL, as it was noted to be present in early stages and persisted through later stages of the disease. Although we do not have the microarray analysis on initial presentation for our patient, microarray-based gene expression profiling done at later stages of the disease did not show presence of gain of 1p36.33-p22.1. Rather a loss at 1p36.33-p36.32 was noted in addition to multiple other genetic abnormalities along with retention of CDKN2A deletion and three intact copies of ABL1 +9q34, present at initial presentation (Figure 2). Therefore, based on different sets of genetic mutations seen in these two cases, we cannot make a conclusion of any specific genetic mutation to be associated with PC-T-LBL.

Treatment of LBL with regimens typically used for non-Hodgkin lymphoma is associated with poor outcomes with only 58% complete remission (CR) rate and a 5-year disease-free survival (DFS) rate of 36% [18]. ALL-based regimens provide a better long-term outcome with CR rates between 55% and 100% and 5-year DFS rates between 45% and 65% [18]. Because of high risk of relapse, hematopoietic stem cell transplantation is often considered as consolidative/potentially curative strategy in first remission or at relapse [19]. However, the exact role of transplantation in T-LBL is not clear because of paucity of large body of data. Moreover, prognostic indicators that can identify patients who are most likely to benefit from transplant have not been defined.

Our case is extremely rare as we found only one previously reported case of primary cutaneous T-LBL. Vezolli et al. described a case of a 29-year-old female with multiple, red-violaceous ulcerated nodules on the left breast that was initially misdiagnosed as PTL-NOS [5]. Similar to our case, diagnosis was changed to T-LBL after positive immunostaining on the initial and relapsed skin specimen with TdT. This highlights the importance of keeping lymphoblastic lymphoma on the differential diagnosis of cutaneous lymphoma like lesions. Staining with TdT and/or other immature T-cell markers should be performed to avoid misdiagnosing T-LBL as other high-grade cutaneous T-cell lymphomas. We believe that this presentation of T-LBL is underreported, and many patients are likely misdiagnosed as having high-grade cutaneous T-cell lymphomas and inappropriately treated with non-Hodgkin's lymphoma- (NHL-) type regimens. Delay in diagnosis and inappropriate treatment will adversely affect the outcome of this aggressive disease. With this case report and literature review, we therefore would like to increase awareness of cutaneous only presentation of T-LBL.

## Figures and Tables

**Table 1:** T-cell lymphoblastic lymphomas with cutaneous involvement reported in the literature.

Source	Sex/age	Cutaneous location	Extracutaneous involvement	Immunohistochemical profile	Treatment	Disease status at last follow-up
Nascimbeni et al. [13]	M/66y	Scalp and trunk	BM and retroperitoneal LN	CD2, CD7, CD3, CD99, TdT, C-kit	Four cycles of CHOP with intrathecal MTX	Complete remission
Ginoux et al. [14]	M/75y	Right arm and occipital area	Bilateral LN on axillary, cervical, and inguinal areas, BM	CD3, CD5, CD4, Ki67, TdT	CHOP	Died 8 Mo after diagnosis
Lee et al. [2]	M/16y	Subcutaneous nodule on the neck	BM, LN	CD99, CD3, CD45RO, TdT	Unspecified chemotherapy	Died 2 y after diagnosis
Lee et al. [2]	M/20y	Multiple scalp nodules	Prostate	CD3, CD4, CD8, CD10, CD99, CD45RO, TdT	Unspecified chemotherapy	Died 5 Mo after diagnosis
Lee et al. [2]	M/25y	Multiple subcutaneous nodules on face, scalp, chest wall	BM, LN, scrotal involvement	CD3, CD10, CD99, CD45RO, TdT	Unspecified chemotherapy	Complete remission 8 y after diagnosis
Lee et al. [2]	M/17y	Subcutaneous nodules on neck and abdomen.	Mediastinal mass, BM, LN	CD3, CD99, CD45RO, TdT.	Unspecified chemotherapy	Complete remission 9 y after diagnosis
Lee et al. [2]	F/25y	Multiple subcutaneous nodules on right breast, neck, chest wall, legs	BM, LN	CD3, CD4, CD8, CD33, CD99, TdT	Unspecified chemotherapy	Relapse
Lee et al. [2]	M/39y	Multiple nodules on scalp	Mediastinal mass, LN, BM, left kidney, pleura	CD3, CD4, CD8, CD10, CD1a, TdT	Unspecified chemotherapy	Complete remission 3 y after diagnosis
Lee et al. [2]	M/22y	Multiple subcutaneous nodules on back	Mediastinal mass, LN, tonsil, nasopharynx	CD3, CD8, CD10, CD45RO, TdT	Unspecified chemotherapy	Partial remission
Vezzoli et al. [5]	F/29y	Multiple nodules on face, breast, thoracomammary region, abdomen, thighs	Primary cutaneous with axillary LN, 23 Mo after diagnosis	CD3, CD5, CD7, CD99, CD45RA, CD79a, KI67, TdT	DHAP + pro MACE cytaBOM + ABSCT	Died after 28 Mo diagnosis
Yaar et al. [15]	M/27y	Cutaneous nodule right cheek and forehead	Mediastinal mass, BM	CD3, CD5, CD7, CD45, CD34, Ki-67	Not described	Not described
Prasad et al. [16]	M/5y	Noduloulcerative purplish lesions on abdominal wall	St Judes stage III unspecified extracutaneous involvement	CD3, CD45, TdT	Not described	Not described
Chimenti et al. [17]	M/65y	Abdomen and extremities	BM	CD1a, CD3, CD43, CD99, TdT	Not described	Died 2 Mo after diagnosis
Sander et al. [9]	F/24y	Malar region, anterior chest wall, breasts	Submental LN, liver, BM	CD2, CD4, CD7, CD10, CD13, TdT	Cyclophosphamide, cytosine, arabinoside, teniposida, deoxycoformycin, vincristine	Died 21 Mo after diagnosis
Sander et al. [9]	M/25y	Scalp	Mediastinal mass, cervical, supraclavicular, axillary LN	CD1, CD2, CD3, CD4, CD5, CD7, CD8, CD10, TdT	Whole brain XRT, intrathecal MTX, cyclophosphamide, doxorubicin, vincristine, L-asparaginas, mercaptopurine	Complete remission

M: male; F: female; Mo: months; y: years; LN: lymph node; BM: bone marrow; MTX: methotrexate; XRT: radiation therapy; DHAP: cytosine arabinoside, cisplatin, and dexamethasone; ProMACE-cytaBOM: cyclophosphamide, methotrexate, vincristine, etoposide, epirubicin, bleomycin, and cytarabine; ABSCT: autologous bone marrow stem cell transplant.

## References

[1] Jabbour E. J., Faderl S., Kantarjian H. M. (2005). Adult acute lymphoblastic leukemia. *Mayo Clinic Proceedings*.

[2] Lee W. J., Moon H. R., Won C. H. (2014). Precursor B- or T-lymphoblastic lymphoma presenting with cutaneous involvement: a series of 13 cases including 7 cases of cutaneous T-lymphoblastic lymphoma. *Journal of the American Academy of Dermatology*.

[3] Maitra A., McKenna R. W., Weinberg A. G., Schneider N. R., Kroft S. H. (2001). Precursor B-cell lymphoblastic lymphoma. *American Journal of Clinical Pathology*.

[4] Millot F., Robert A., Bertrand Y. (1997). Cutaneous involvement in children with acute lymphoblastic leukemia or lymphoblastic lymphoma. *Pediatrics*.

[5] Vezzoli P., Novara F., Fanoni D. (2012). Three cases of primary cutaneous lymphoblastic lymphoma: microarray-based comparative genomic hybridization and gene expression profiling studies with review of literature. *Leukemia and Lymphoma*.

[6] Siegel R. L., Miller K. D., Jemal A. (2018). Cancer statistics, 2018. *CA: A Cancer Journal for Clinicians*.

[7] Esparza S. D., Sakamoto K. M. (2005). Topics in pediatric leukemia—acute lymphoblastic leukemia. *MedGenMed*.

[8] Schmitt I. M., Manente L., Di Matteo A., Felici F., Giangiacomi M., Chimenti S. (2009). Lymphoblastic lymphoma of the pre-B phenotype with cutaneous presentation. *Dermatology*.

[9] Sander C. A., Medeiros L. J., Abruzzo L. V., Horak I. D., Jaffe E. S. (1991). Lymphoblastic lymphoma presenting in cutaneous sites. *Journal of the American Academy of Dermatology*.

[10] Marks D. I., Paietta E. M., Moorman A. V. (2009). T-cell acute lymphoblastic leukemia in adults: clinical features, immunophenotype, cytogenetics, and outcome from the large randomized prospective trial (UKALL XII/ECOG 2993). *Blood*.

[11] Sekimizu M., Sunami S., Nakazawa A. (2011). Chromosome abnormalities in advanced stage T-cell lymphoblastic lymphoma of children and adolescents: a report from Japanese Paediatric Leukaemia/Lymphoma Study Group (JPLSG) and review of the literature. *British Journal of Haematology*.

[12] Schraders M., van Reijmersdal S. V., Kamping E. J. (2009). High-resolution genomic profiling of pediatric lymphoblastic lymphomas reveals subtle differences with pediatric acute lymphoblastic leukemias in the B-lineage. *Cancer Genetics and Cytogenetics*.

[13] Nascimbeni C., Chantepie S., Brugiere C., Comoz F., Salaun V., Verneuil L. (2017). Cutaneous involvement in T-lymphoblastic lymphoma. *Annales de Dermatologie et de Vénéréologie*.

[14] Ginoux E., Julia F., Balme B., Thomas L., Dalle S. (2015). T-lymphoblastic lymphoma with cutaneous involvement. *World Journal of Clinical Cases*.

[15] Yaar R., Karen R., Mahalingam M. (2010). When dead cells tell tales-cutaneous involvement by precursor T-cell acute lymphoblastic lymphoma with an uncommon phenotype. *American Journal of Dermatopathology*.

[16] Prasad P. G., Cyriac S., Sagar T. G., Rathnam K. (2010). T-lymphoblastic lymphoma relapsing in skin—a rare clinical scenario. *Indian Journal of Hematology and Blood Transfusion*.

[17] Chimenti S., Fink-Puches R., Peris K. (1999). Cutaneous involvement in lymphoblastic lymphoma. *Journal of Cutaneous Pathology*.

[18] Cortelazzo S., Ponzoni M., Ferreri A. J. M., Hoelzer D. (2011). Lymphoblastic lymphoma. *Critical Reviews in Oncology/Hematology*.

[19] Lepretre S., Graux C., Touzart A., Macintyre E., Boissel N. (2017). Adult T-type lymphoblastic lymphoma: treatment advances and prognostic indicators. *Experimental Hematology*.

